# Pregnancy weight gain and breast cancer risk

**DOI:** 10.1186/1472-6874-4-7

**Published:** 2004-10-21

**Authors:** Tarja I Kinnunen, Riitta Luoto, Mika Gissler, Elina Hemminki, Leena Hilakivi-Clarke

**Affiliations:** 1 Tampere School of Public Health, 33014 University of Tampere, Finland; 2 UKK Institute, PL 30, 33501 Tampere, Finland; 3 National Research and Development Centre for Welfare and Health, PL 220, 00531 Helsinki, Finland; 4 Lombardi Cancer Center and Department of Oncology, Georgetown University, 3970 Reservoir Rd, NW, Washington, DC 20057, USA

## Abstract

**Background:**

Elevated pregnancy estrogen levels are associated with increased risk of developing breast cancer in mothers. We studied whether pregnancy weight gain that has been linked to high circulating estrogen levels, affects a mother's breast cancer risk.

**Methods:**

Our cohort consisted of women who were pregnant between 1954–1963 in Helsinki, Finland, 2,089 of which were eligible for the study. Pregnancy data were collected from patient records of maternity centers. 123 subsequent breast cancer cases were identified through a record linkage to the Finnish Cancer Registry, and the mean age at diagnosis was 56 years (range 35 – 74). A sample of 979 women (123 cases, 856 controls) from the cohort was linked to the Hospital Inpatient Registry to obtain information on the women's stay in hospitals.

**Results:**

Mothers in the upper tertile of pregnancy weight gain (>15 kg) had a 1.62-fold (95% CI 1.03–2.53) higher breast cancer risk than mothers who gained the recommended amount (the middle tertile, mean: 12.9 kg, range 11–15 kg), after adjusting for mother's age at menarche, age at first birth, age at index pregnancy, parity at the index birth, and body mass index (BMI) before the index pregnancy. In a separate nested case-control study (n = 65 cases and 431 controls), adjustment for BMI at the time of breast cancer diagnosis did not modify the findings.

**Conclusions:**

Our study suggests that high pregnancy weight gain increases later breast cancer risk, independently from body weight at the time of diagnosis.

## Background

Sensitivity of the breast to hormones and environmental exposures varies throughout a woman's life span [1]. During pregnancy, the breast undergoes extensive changes in preparation for lactation. High estrogenicity during pregnancy causes marked cellular proliferation, in both in the normal and tumor cells. Normal breast cells differentiate to milk-secreting alveoli, while tumor cells, if present, continue to multiple and eventually form a detectable tumor. These two events probably explain the dual effect of pregnancy on breast cancer risk: pregnancy before age 20 reduces, whereas first pregnancy after age 30 increases, breast cancer risk [2]. In young women, pregnancy may eliminate future targets for neoplastic changes by differentiating target cells [3]; the breast tissue of older first time mothers is more likely to have acquired malignant cells that are stimulated by high pregnancy hormonal environment.

Women whose pregnancy estrogen levels are elevated are at an increased risk of breast cancer. For example, women who took the synthetic estrogen diethylstilbestrol (DES) during pregnancy are at an increased risk of developing breast cancer [4], as are women who suffered from severe pregnancy nausea [5] or who gave birth to heavy newborns [6]. Both nausea in pregnancy and high birth weight are linked to elevated pregnancy estrogen levels [7,8] Conversely, pregnant women having high alpha feto-albumin levels [9,10], or suffering from hypertension or pre-eclampsia [11,12], exhibit a reduced risk. Alpha feto-protein has direct antiestrogenic activity and binds estrogens, reducing their biological availability [13,14]. Hypertension during pregnancy is linked to reduced estrogen and increased testosterone levels [15]. A recent study in which estrogen levels were measured in stored blood samples of pregnant women later diagnosed with breast cancer, provides direct evidence in support of high estrogen and low progesterone levels in increasing maternal breast cancer risk [16]. However, some studies have failed to find an association between pregnancy estrogen levels, determined indirectly, and maternal breast cancer risk [11,17].

Adipose tissue aromatizes androgens to estrogens, and thus high body mass index (BMI) is linked to elevated estrogen levels in postmenopausal women [18]. Some studies suggest that high pregnancy weight gain may be associated with increased pregnancy estrogen levels [19], although this has not been confirmed in more recent studies [20,21]. The goal of this study was to determine whether high pregnancy weight gain affects breast cancer risk.

## Methods

### The cohort

The study population was a historic cohort of women pregnant between 1954 and 1963 in Helsinki, Finland (n = 4,090). The cohort was a sample gathered for a study on hormone exposure, including 2,022 exposed, 2,062 controls and 6 women with unknown hormone exposure status. Information on the cohort was collected from the maternity cards of municipal maternity centers, which are used by most pregnant Finnish women. The hormone-exposed women had been prescribed estrogen or progestin drugs during pregnancy to prevent early abortion or preterm delivery. For each exposed woman, a woman next in the maternity center file who gave birth during the same year and had not been prescribed hormones during pregnancy, was chosen as a control. The cohort has been previously prescribed in detail [22,23]. There were no differences in breast or other estrogen-dependent cancers between hormone-exposed and control mothers [22]. Visits to a private doctor were used as an indicator of socio-economic status, since no information on education or occupation at the time of the index pregnancy was available.

Cancer cases were identified through a record linkage to the national cancer registry until June 2001. Mortality and emigration data were obtained from the population registry until August 2001. The linkage between the cohort and the registries was based on a unique personal identification number.

### Inclusions and exclusions

**Figure 1:**
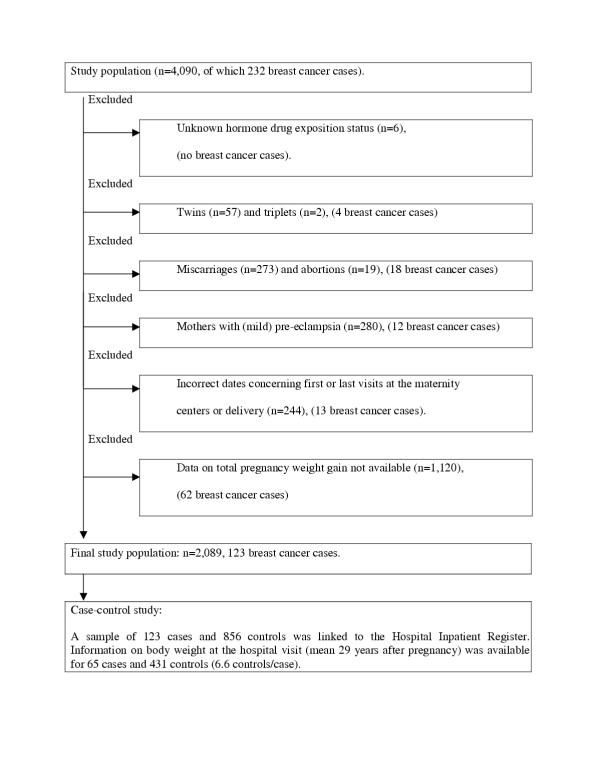
Study population and exclusions.

Inclusion criteria were the following: first and last visit at the maternity center between 4–45^th ^gestation weeks, the time between the body weight measurements 3–300 days, and delivery between 22–45^th ^gestation weeks. For each mother, the gestation week she gave birth was determined by using the date of estimated timing of delivery. Women who did not fulfill these criteria were excluded (Fig. 1). In addition, women with multiple births were excluded because their weight gain is not comparable to that of mothers of singletons. Mothers with pre-eclampsia or eclampsia were excluded because they accumulate weight as fluid retention during pregnancy, and have been reported to have a reduced breast cancer risk [11,12].

Pregnancy weight gain was first calculated as the difference between the first and last visit to maternity center. However, this window varied considerably among included mothers (range 3–295 days). The time-period of calculated weight gain was therefore adjusted by extrapolating a line for each mother to reflect her weight increase during pregnancy. The calculations are described in detail in Additional File 1. After the unstable period of early pregnancy, a mother's weight increases linearly [24]. Mothers usually begin to gain weight after the first trimester (e.g [25]). We extrapolated the line separately for 0–15^th ^(Line A) and 15–40^th ^gestation weeks (Line B) for each mother. Weight gain was extrapolated to continue until 40^th ^gestation week for all mothers, although 22.2% of mothers delivered at 39^th ^gestation week or before.

For mothers for whom both Line A (n = 2,143) and Line B (n = 2,184) were available, total pregnancy weight gain was calculated by adding the extrapolated weight gains from both periods. Thus, total pregnancy weight gain could be extrapolated only for 2,089 women. Cases and controls (66.5% vs 65.0%) did not differ concerning the number of available weight measurements. For the rest of the women, either the first weight measurement was later than 24^th ^gestation week, the last weight measurement was before 30^th ^gestation week, or information on pre-pregnancy weight, weight at the first or the last visit, or timing of the visits was not available. As indicated above, these subjects were excluded from the analyses.

We compared the characteristics of the mothers who were excluded (n = 2,001) to the characteristics of the mothers in the final study population for whom total pregnancy weight gain could be determined (n = 2,089). The two groups were similar in regard to breast cancer incidence, age at menarche, height and the frequency of visits to a private doctor. However, the excluded mothers were older (mean: 27.1 years vs. 26.5 years, p < 0.001), heavier (58.7 kg vs. 57.3 kg, p < 0.001; body mass index, BMI: 22.3 kg/m^2 ^vs. 21.8 kg/m^2^, p < 0.001), older at first birth (25.2 years vs. 24.7 years, p = 0.016), had more children during index pregnancy (1.92 vs. 1.81 at index birth, p < 0.001), and were more often exposed to estrogen or progestin drugs (50.3% vs. 48.6%, p = 0.021). Their children were shorter (mean 49.3 cm vs. 50.3 cm, p < 0.001) and weighed less (mean 3,310 g vs. 3,472 g, p < 0.001), suggesting that excluded mothers' pregnancy weight gain might have been lower. It is probable that exclusion of these women had no major effect on the findings.

### The case-control study

A nested case-control study was performed to determine whether later weight development confounded the association between pregnancy weight gain and breast cancer risk. A sample of women was chosen from the final cohort (n = 2,089) that included all breast cancer cases with data on pregnancy weight gain (n = 123). For each case, we chose seven randomly selected controls (n = 856) who were born in the same year as the case. These 979 women were linked to the Hospital Inpatient Registry to obtain information on the women's stays in hospitals. 117 cases were identified with a hospital visit in average 0.4 months after breast cancer diagnosis (median 0.0, range from -16.3 to 17.2), and of these cases information on body weight and height was available for 65 (53% of 123 cases). Among the controls, 699 had been a patient in a hospital at a similar age than their respective cases (maximum difference +/-5 years), and 431 of them had weight and height available in the hospital archives (50% of 856 controls, 6.6 controls/case).

The breast cancer cases with no information on later body weight did not differ from the cases used for the nested case-control analysis. The controls with no information on later body weight were approximately 1.5 years older at the time of the hospital visit than the controls included to the study (p = 0.027).

### Statistical analysis

Statistical significance of possible differences in baseline characteristics of the study population, pregnancy weight gain and postpartum weight loss and weight retention by tertiles of pregnancy weight gain was tested by using analysis of variance for continuous variables and χ^2^-test for proportions. The incidence of breast cancer per 100,000 person years was counted by groups of 5 kg pregnancy weight gain, tertiles of pregnancy weight gain and tertiles of postpartum weight retention. Person years were calculated from the delivery to the diagnosis of breast cancer or other endpoint including death, emigration or end of the study.

The association between pregnancy weight gain and breast cancer risk was further examined using a Cox regression model. Total pregnancy weight gain was included as a categorical covariate (tertiles) in the model. Age at menarche, age at first birth, age at index pregnancy, BMI before pregnancy, and parity (at index birth) were all used as continuous covariates in the model. Postpartum weight retention 51 days after delivery (mean) was later added to the model.

The incidence of breast cancer was counted and the Cox regression model was carried out also separately for pre- and postmenopausal breast cancers. Information on the age at menopause was not available. Therefore all women were expected to have menopause at the age of 50 years.

In the case-control study, weight and BMI change between pre-pregnancy and at the time of later hospital visit were compared between the tertiles of pregnancy weight gain (analysis of variance). A Cox regression model that included later BMI was also used to analyze the data.

## Results

In the cohort, 123 (5.9%) women developed breast cancer during the mean follow-up of 38.9 years. The mean age at diagnosis was 56.0 years (range 35–74). Background characteristics and index pregnancies are described by tertiles of pregnancy weight gain in Table 1. Low pregnancy weight gain (<11 kg) was associated to slightly higher ages during index pregnancy, during first pregnancy and at menarche, and to lower height, higher BMI before pregnancy, higher gestation weeks at delivery, lower weight of the placenta, smaller infant and lower proportion of users of estrogen drugs compared to women with higher pregnancy weight gain (11–15 kg or >15 kg).

**Table 1:** Background characteristics of index pregnancy, by tertiles of estimated pregnancy weight gain. Means (and SD) or percentiles are shown.

	Pregnancy weight gain (kg)			
		
	< 11 (n = 696)	11–15 (n = 697)	>15 (n = 696)	p-value
*Background*				
Mother's age (year)^1^	27.0 (5.3)	26.2 (4.9)^2^	26.2 (5.0)^2^	0.006
Mother's age at first birth (years)	25.2 (4.8)	24.4 (4.4)^2^	24.5 (4.4)^2^	0.003
Married (%)^1^	97	98	98	0.720
Visits to a private doctor (%)^1^	49	45	51	0.053
Mother's height (cm)	161.2 (5.5)	162.0 (5.2)^2^	162.9 (5.2)^23^	<0.001
Mother's body mass index before pregnancy (kg/m^2^)	22.3 (2.9)	21.6 (2.3)^2^	21.6 (2.5)^2^	<0.001
Mother's age at menarche (year)	14.2 (1.6)	13.9 (1.6)^2^	13.8 (1.6)^2^	<0.001
Regular menstrual cycles (%)	94	93	95	0.296
Parity (at index birth)	1.8 (1.1)	1.8 (1.0)	1.8 (1.1)	0.896
*Index pregnancy*				
Gestation weeks at delivery (week)	40.6 (2.2)	40.4 (2.1)	40.2 (2.2)^2^	<0.001
Exposed to estrogens (%)	45	49	52	0.040
Weight of the placenta (g)^4^	603 (112)	634 (124)^2^	661(205)^23^	<0.001
Infant height (cm)^4^	50.0 (2.3)	50.3 (2.1)	50.6 (2.5)^23^	<0.001
Infant weight (g)	3,376 (514)	3,466 (504)^2^	3,577(544)^23^	<0.001
Low birth weight (%)	5	3	3	0.305

^1 ^at the time of index pregnancy

^2 ^a statistically significant difference compared to the lowest tertile (<11 kg)

^3 ^a statistically significant difference compared to the middle tertile (11–15 kg)

^4 ^total n = 2,055–2,089, except for placental weight (n = 1,217) and infant height (n = 2,006)

Weight development during and after pregnancy is presented by tertiles of pregnancy weight gain in Table 2. Higher weight gain during pregnancy was associated to higher weight loss after delivery, but also to higher weight retention and BMI at the postpartum check-up visit.

**Table 2:** Weight gain during and after pregnancy by tertiles of estimated pregnancy weight gain. Means (and 95% confidence intervals) are shown.

	Pregnancy weight gain (kg)			
	<11 (n = 696)	11–15 (n = 697)	>15 (n = 696)	p-value

*Mother's weight gain (kg)*				
Total weight gain, weeks 0–40	8.6 (8.5–8.8)	12.9 (12.9–13.0)	18.2 (18.0–18.4)	<0.001
*Weight after delivery*^1^				
Weight change from 40^th ^week (kg)	-7.4 (-7.7 – -7.2)	-8.8 (-9.0 – -8.6)	-10.6 (-10.9 – -10.3)	<0.001
Weight compared to pre- pregnancy weight (kg)	+1.3 (1.0–1.6)	+4.1 (3.8–4.3)	+7.6 (7.3–8.0)	<0.001
BMI (kg/m^2^)	22.6 (22.4–22.9)	23.2 (22.9–23.4)	24.4 (21.1–24.6)	<0.001
*Weight at the hospital visit*^2^	(n = 167)	(n = 170)	(n = 159)	
Change from pre-pregnancy weight (kg)	+ 6.4 (5.1–7.8)	+10.4 (9.0–11.9)	+12.5 (10.8–14.3)	<0.001
BMI (kg/m^2^)	25.0 (24.3–25.6)	25.6 (25.0–26.2)	26.3 (25.6–27.0)	0.021
Change from pre-pregnancy BMI (kg/m^2^)	+ 2.4 (1.9–3.0)	+ 4.0 (3.4–4.6)	+ 4.8 (4.1–5.4)	<0.001

^1 ^on postpartum day 51, range 40–78, total n = 1,314–1,713

^2 ^29 years after pregnancy in average, range 9–47

### Breast cancer incidence per 100,000 person years

The mean BMI before pregnancy was 21.8 kg/m^2 ^and the mean total extrapolated weight gain during pregnancy was 13.3 kg (range -5.0–33.1 kg) in our cohort. Average pregnancy weight gain (and range) was 13.1 kg (-3.0–33.1) among primiparas, 13.5 kg (1.9–30.7) among women who gave birth to their second child and 13.2 kg (-5.0–32.4) among women who gave birth to at least their third child. The incidence of breast cancer by 5 kg categories of total pregnancy weight gain is shown in Table 3. Higher pregnancy weight gain was associated with a higher incidence of breast cancer. However, the number of women in some of the weight gain categories was small, and therefore the statistical analyses were carried out in tertiles of total pregnancy weight gain (Table 4). The incidence of breast cancer was significantly higher in mothers in the highest tertile of pregnancy weight gain (15–33 kg), when compared to the middle tertile (11–15 kg) (p = 0.04). Breast cancer incidence was lowest in the middle tertile, but no differences in the risk were seen in the mothers of the lowest tertile of weight gain (less than 11 kg), when compared with the other two categories.

**Table 3:** Breast cancer incidence (per 100,000 person years, py) by estimated total weight gain (weeks 0–40).

Weight gain (kg)	Breast cancer cases (n)	Number of women	Py 171	Incidence 0
<0	0	4		
0–4.99	1	42	1,677	60
5–9.99	23	423	16,614	138
10–14.99	53	954	37,266	142^1^
15–19.99	33	508	19,498	169
≥20	13	158	6,086	213
total	123	2,089	81,312	151

^1 ^Breast cancer incidence was exceptionally high (200 per 100,000) in mothers who gained 10–10.99 kg during pregnancy.

**Table 4:** Incidence (per 100,000 person years) and unadjusted and adjusted rate ratios (RR)^1 ^and confidence intervals (CI) on the Cox model for breast cancer by tertiles of estimated total weight gain (kg) in pregnancy (weeks 0–40), and by tertiles of postpartum weight retention.

	Breast cancer cases	Number of women	Incidence	p-value	Unadjusted RR (95% CI)	Adjusted RR (95% CI)^1^
*Pregnancy weight gain (kg)*				0.09		
<11	39	696	143		1.18 (0.74–1.88)	1.11 (0.68–1.83)
11–15	33	697	121		1.00 (ref.)	1.00 (ref.)
> 15	51	696	190		1.59 (1.03–2.47)	1.62 (1.03–2.53)
*Postpartum weight retention (kg)^2^*				0.33		
<3	26	558	121		1.00 (ref.)	1.00 (ref.)
3–5	35	539	170		1.29 (0.72–2.34)	1.36 (0.73–2.54)
>5	35	545	170		1.54 (0.87–2.74)	1.56 (0.85–2.86)
*Pregnancy weight gain (kg), case-control study^3^*						
<11	19	167	-	-	1.01 (0.54–1.91)	0.95 (0.49–1.84)
11–15	19	170	-	-	1.00 (ref.)	1.00 (ref.)
> 15	27	159	-	-	1.50 (0.83–2.69)	1.48 (0.81–2.69)

^1 ^Adjusted for age at menarche, age at first birth, age at index pregnancy, parity (at index birth) and body mass index (BMI) before pregnancy. For the RR for postpartum weight retention, adjusted also for pregnancy weight gain (weeks 0–40).

^2 ^Weight on postpartum day 51, range 40–78, compared to pre-pregnancy weight.

^3 ^Adjusted for age at menarche, age at first birth, age at index pregnancy, parity (at index birth), BMI before pregnancy, and BMI during the hospital visit in average 29 years after pregnancy.

All analyses were initially carried out separately for pre- and postmenopausal breast cancers. The results for postmenopausal women were similar to the results for the whole cohort (results not shown). The incidence of premenopausal breast cancer was too low for statistical analysis and pre- and postmenopausal breast cancers were not separated further in the analyses. When these analyses were restricted to mothers who delivered after 39^th ^gestation week, results were similar than in the whole cohort (results not shown).

The incidence of breast cancer was calculated separately for early (0–15^th ^gestation weeks) and later pregnancy weight gain (15–40^th ^gestation weeks). Early pregnancy weight gain was not associated with breast cancer risk. The impact of later pregnancy weight gain was similar to the impact of total weight gain, but more modest (results not shown).

### Multivariate analysis

Both unadjusted and multivariate adjusted rate ratios and confidence intervals for the risk of breast cancer are presented in Table 4. In the Cox regression model, mothers in the highest tertile of pregnancy weight gain (>15 kg) had a 1.62-fold higher risk for breast cancer compared to mothers in the middle tertile (average weight gain 12.9 kg), when age at menarche, age at first birth, age at index pregnancy, BMI before pregnancy and parity at index birth were included in the model. To assess the sensitivity of these analyses, the lowest and highest weight gain groups were used as reference groups. When the lowest weight gain group was the reference group, no differences among the groups were seen. However, when the highest weight gain group was the reference group, women with average weight gain had significantly lower risk of breast cancer (multivariate adjusted RR 0.62, 95% CI 0.40–0.97).

When the middle tertile of weight gain was again used as the reference group and the analysis was restricted to mothers who delivered after 39th week of gestation, the results were essentially similar although statistically not significant (data not shown). The results did not either change when adjusted additionally for the year of index birth. The increased breast cancer risk in the highest tertile of pregnancy weight gain was found only for postmenopausal breast cancer (relative risk, RR = 1.80, 95% confidence interval, CI 1.05–3.07, p = 0.03). The RR for premenopausal cancer was 1.00 (95% CI 0.40– 2.48, p = 0.99). However, the number of premenopausal breast cancer cases with the information on all variables in the model was too low (n = 25) to yield sufficient power.

No statistically significant differences in breast cancer risk were observed between the tertiles of postpartum weight retention, determined approximately 51 days after delivery (Table 4).

### Other results

Later age at menarche was marginally related to a decreased risk of breast cancer (adjusted RR = 0.99, 95% CI 0.97–1.00). Mother's age at the time of first pregnancy or at the index pregnancy, parity at index birth or BMI before pregnancy were not statistically significantly associated with the risk of breast cancer. The results were similar when height and weight were used as separate variables in the model, instead of BMI.

Lower pre-pregnancy BMI was associated with higher weight gain during pregnancy (p < 0.001) and higher postpartum weight retention (p = 0.003), but not with postpartum weight loss. The differences in the incidence of breast cancer were not statistically significant between the pre-pregnancy BMI-categories.

### The case-control study

Women who gained at least 15 kg weight during pregnancy had a higher BMI at the time of later hospital visit (29 years after pregnancy in average) than women who gained <11 kg weight during pregnancy (p = 0.021) (Table 2.). Changes in body weight (p < 0.001) and BMI (p < 0.001) were also higher in women who gained 11–15 kg or >15 kg compared to women who gained <11 kg during pregnancy. These findings are in agreement with earlier reports showing a link between excessive pregnancy weight gain and becoming overweight/obese later on [26,27].

The time window between pregnancy and assessment of BMI during later hospital visit was similar among the tertiles of pregnancy weight gain (29.2 vs. 30.0 vs. 30.1 years, p = 0.397). In the Cox regression model, women's later BMI at the time of diagnoses was not associated with breast cancer risk (adjusted RR = 0.96, 95% CI 0.90–1.04). Further, results relating to pregnancy weight gain and breast cancer risk were not altered by adding data on later BMI to the model (Table 4). It is to be noted that since fewer women were included to this analysis, the effect of pregnancy weight gain did not reach statistical significance.

## Discussion

The results obtained in our study indicate that higher than recommended pregnancy weight gain increased mothers' risk of developing breast cancer. Thus, women who gained more than 15 kg during pregnancy had a 62% increase in breast cancer risk, compared to those who gained between 11–15 kg. The Institute of Medicine (IOM) published their most recent recommendations for pregnancy weight gain in 1990 [28]. The recommended pregnancy weight gain in the USA is 11.5–16 kg for women with normal pre-pregnancy BMI; i.e., they are not obese or underweight. Pregnancy weight gain recommendations are lower (7–11.5 kg) for overweight women and higher (12.5–18 kg) for underweight women. As seen in Table 3, the incidence of breast cancer in our study was highest among women who gained more than 20 kg during pregnancy, suggesting that the increase in risk may apply primarily to women at the most extreme range of pregnancy weight gain.

An increase in breast cancer risk was seen mostly in women who were diagnosed with this disease after age 50 and thus were postmenopausal. However, the number of premenopausal breast cancers was low in the cohort, and we cannot exclude the possibility that pregnancy weight gain may also increase the risk of premenopausal breast cancer.

Data generated in epidemiological studies rarely provide causal relationships. We propose four different mechanisms that may link high pregnancy weight gain to a later increase in breast cancer risk. First, weight retention in women who gained excessive amounts of weight during pregnancy may have persisted into their postmenopausal years. Women prone to postpartum weight retention might also be prone to long-lasting weight gain after pregnancy [29], and high BMI during postmenopausal years increases breast cancer risk [26]. To examine this possibility, information on body weight at the time of breast cancer diagnosis was obtained. If the association between pregnancy weight gain and breast cancer risk was affected by later weight development, breast cancer cases should have had higher BMI at the time of diagnosis. As this was not the case, we propose that high pregnancy weight gain increases breast cancer risk independently from body weight at the time of diagnosis.

Another alternative is that women who gained an excessive amount of weight during pregnancy may have had higher pregnancy hormone and growth factor levels than women who gained within the recommended range, stimulating the growth of existing malignant cells in the breast, leading to development of a detectable tumor. Several studies have shown that markers of high pregnancy estrogen levels increase mother's breast cancer risk [4-6,9-12,16]. Estrogen levels may correlate with high pregnancy weight gain [19], but two recent studies have not confirmed this observation [20,21]. Other possible hormones that could be mediating the effect of pregnancy weight gain on breast cancer risk include leptin. Leptin levels correlate strongly with BMI [27], also during pregnancy [30], and leptin is suggested to increase breast cancer risk [31]. We did not have any biological samples available for hormone measurements.

It is known that high hormone levels increase the proliferation of normal breast cells that then is accompanied by increased genomic instability and accumulation of DNA adducts [16,32]. Thus, the third explanation is that high pregnancy weight gain increased the likelihood of DNA damage and mutations in genes that initiate breast cancer. Since the window between index pregnancy and diagnosis of breast cancer was approximately 30 years, there was enough time for the initiation to have taken place during pregnancy.

Finally, known and unknown causes of breast cancer may have confounded the results. For example, these causative factors might be more common in women who gain excessive amounts of weight during pregnancy or they caused women to gain excessive amounts of weight during pregnancy. A theoretical example is a gene mutation/polymorphism that could both make a woman more prone to gain weight during pregnancy and increases breast cancer risk.

Methodological limitations have to be considered when interpreting the results, and they include high rate of exclusion and an exposure to estrogenic drugs during pregnancy. Of the 4,090 women available for the study, 48.9 % were excluded for reasons listed in Figure 1 (109 of which were diagnosed with breast cancer). Total pregnancy weight gain could be extrapolated only for 2,089 women, of which 123 had developed breast cancer. Other information on background and index pregnancies indicated that the excluded women might have gained less weight during pregnancy than the final study population (see chapter Inclusions and exclusions). However, we found no evidence that breast cancer incidence was different between the women excluded and included to the study.

Some women in our cohort had been exposed to synthetic estrogens during pregnancy to avoid a threatening miscarriage, and this exposure might have affected the results. However, it was the initial reason for obtaining information from pregnant women, and we found no effect of the drug exposure on the incidence of breast cancer. We are not aware of any other cohort that could be used to assess the link between pregnancy weight gain and breast cancer risk, but if such a cohort becomes available, and it is not potentially compromised by high rate of exclusion of subjects or an exposure to drugs during pregnancy, the present results can be either confirmed or nullified. Follow-up of "old" cohorts similar to ours is rarely possible, making our study relatively unique.

Another area of potential source for errors is the variability in time period between the weight measurements during pregnancy (range 3–295 days), requiring us to extrapolate the pregnancy weight gain for each woman. This step also has been successfully used in other studies [33]. A further weakness of the study was that no information on weight gain in previous and subsequent pregnancies was available. Therefore, we cannot exclude the possibility that a woman who did not develop breast cancer and during the index pregnancy gained less than 15 kg, might have had subsequent pregnancies that were characterized by excessive weight gain.

Finally, in the case-control study that determined the impact of body weight at the time of diagnosis on breast cancer risk, information on this weight was obtained only for 53% of the cases and 50% of the controls. However, the direction of bias rising from exclusion may have diluted the effect, rather than caused it.

In conclusion, our findings suggest that excessive pregnancy weight gain increased later risk of developing breast cancer. This association needs to be further confirmed in prospective studies.

## List of abbreviations

diethylstilbestrol – DES; body mass index – BMI

## Competing interests

The author(s) declare that they have no competing interests.

## Authors' contributions

*TK*: PhD student who collected and put together all the material for the study, managed the data and did statistical analysis, and participated in writing the manuscript.

*MG*: Participated in statistical analysis of the data and writing the manuscript.

*EH*: Collected the original data base of pregnant women and was in charge of linking the data base to cancer registry, participated in statistical analysis planning and writing the manuscript.

*RL*: Generated the idea of testing the hypothesis in the Hemminki data base, and participated in all stages of the study and in writing the manuscript.

*LH-C*: Generated the hypothesis, obtained funding for the study, and was in charge of writing the manuscript.

## Pre-publication history

The pre-publication history for this paper can be accessed here:



## Supplementary Material

Additional File 1 Calculation of line a and line b for each mother. Click here for file
