# Supplementary material for: Pregnancy weight gain and breast cancer risk
Source: BMC Womens Health. 2004 Oct 21;4:7. doi: 10.1186/1472-6874-4-7 (PMC535935; doi:10.1186/1472-6874-4-7)
Supplement: Additional File 1 — Calculation of line a and line b for each mother. [file 1472-6874-4-7-S1.doc]

*Appendix 1.* Calculation of Line A and Line B for each mother

Calculation of pregnancy weight gain is based on self-reported pre-pregnancy weight and weights measured at the first and the last maternity center visits and when the mother arrived at the hospital for delivery. Availability of the data from these measurements is shown below by breast cancer status (n, %).

|  | Pre-pregnancy weight,  n (%) | The first visit at 4-15 week, n (%) | The first visit at 15-24 week, n (%) | The last visit >30 week,  n (%) | At delivery  >30 week,  n (%) | Postpartum  51 days,  n (%) |
| --- | --- | --- | --- | --- | --- | --- |
| Cases (n=185) | 167 (90) | 81 (44) | 62 (34) | 141 (76) | 64 (35) | 97 (52) |
| Controls (n=3,024) | 2,711 (90) | 1,220 (40) | 1,057 (35) | 2,401 (79) | 1,014 (34) | 1,557 (51) |
| Total (n=3,209) | 2,878 (90) | 1,301 (41) | 1,119 (35) | 2,542 (79) | 1,078 (34) | 1,654 (52) |

The formula used to calculate the lines was:

$y=a+bx,\text{ where}$ $b=\frac{w_{2}-w_{1}}{g_{2}-g_{1}}$ and .

For Line A (0-15 gestation week), w_1_= mothers pre-pregnancy weight, w_2_= weight measured before 15^th^ gestation week (or if that was not available, for 967 of 2,143 women (45.1%), abstracted from Line B at 15^th^ gestation week), g_1_= gestation week for w_1_ (0) and g_2_= gestation week for w_2_ (mean 11.3 week).

For Line B (15-40 gestation weeks), w_1_= weight measured between 15-24^th^ gestation week (or if that was not available, for 1,122 of 2,184 women (51.4%), abstracted from Line A at 15^th^ gestation week), w_2_= weight measured after 30^th^ gestation week, g_1_= gestation week for w_1_ (mean 18.2 week) and g_2_= gestation week for w_2_ (mean 39.1 week).

The total pregnancy weight gain for 0-40 gestation weeks (both Line A and Line B) could be extrapolated for 2,089 (65% of 3,209) of the women.

| Extrapolated average pregnancy weight gain (kg) in the cohort: the average Line A (n=2,143) for 0-15^th^ and the average Line B (n=2,184) for 15-40^th^ gestation weeks |
| --- |
